# Challenging clinical cases in HCV infection

**DOI:** 10.1186/1471-2334-14-S5-S7

**Published:** 2014-09-05

**Authors:** Alessio Strazzulla, Giovanni Matera, Selma Valerie Mammone, Vittoria Vaccaro, Vincenzo Pisani, Chiara Costa, Francesco Manti, Patrizia Doldo, Lucio Cosco, Francesco Quintieri, Francesco Cesario, Maria Carla Liberto, Aida Giancotti, Carlo Torti, Alfredo Focà

**Affiliations:** 1 Unit of Infectious Diseases, Department of Medical and Surgical Sciences, University "Magna Graecia", Catanzaro, Italy; 2 Institute of Microbiology, Department of Health Sciences, "Magna Graecia" University of Catanzaro, Catanzaro, Italy; 3 Department of Radiology, "Magna Graecia" University of Catanzaro, Catanzaro, Italy; 4 Centre for Oncology, "Tommaso Campanella" Fundation, Catanzaro, Italy; 5 Unit of Infectious Diseases, "Pugliese-Ciaccio" Hospital, Catanzaro, Italy; 6 Unit of Infectious Diseases, "Annunziata" Hospital, Cosenza, Italy; 7 University Unit of Infectious Diseases, University of Brescia, School of Medicine, Brescia, Italy

**Keywords:** HCC, Hydatidosis, Echinococcosis, HCV, Interferon, Thalassemia, Sickle cell disease

## Abstract

We present clinical cases, which underline some difficulties in diagnosis and treatment of hepatitis C virus (HCV) infection. Case report #1 shows a patient who avoided clinical follow-up for HCV until the development of hepatocellular carcinoma. In this patient, non-invasive procedures did not allow to make a differential diagnosis between hydatidosis and hepatocellular carcinoma but diagnosis was only made with liver biopsy.

In the case report #2, 24-week treatment with peg-interferon α2 and ribavirin was successfully administered to a HCV genotype-1b infected patient. Shortening HCV treatment did not impair sustained virological response, probably because HCV RNA was low (< 200,000 IU/l) at baseline.

Lastly, a case series of patients (#3-6) with hemoglobinopathies is described. Sustained virological response after peg-interferon α2 and ribavirin was achieved in 2 out of 4 patients. While no severe treatment limiting hematological effects were encountered, patients needed more frequent blood transfusions. Thus, new anti-HCV schemes without peg-interferon and ribavirin are urgently needed.

## Case report #1 - Hepatocellular carcinoma or echinococcosis? Considerations from a challenging case of neglected HCV

Alessio Strazzulla, Giovanni Matera, Selma Valerie Mammone, Vittoria Vaccaro, Vincenzo Pisani, Chiara Costa, Francesco Manti, Patrizia Doldo, Maria Carla Liberto, Aida Giancotti, Carlo Torti, Alfredo Focà

## Background

Southern Italy is currently a hyper-endemic area for hepatitis C virus (HCV) infection. Moreover, it has been shown that incidence of genotype 4 (gt-4) is increasing in the Calabria region [1]. These data, however, do not reflect the actual prevalence of infection, because there is a large proportion of cases that are not diagnosed or not retained into care. For 18 years, it has been reported that chronic HCV infection is one major cause of hepatocellular carcinoma (HCC) [2]. Thus, early diagnosis and treatment of HCV are important to prevent this complication. Also, early diagnosis of HCC is important to better control the disease with a less aggressive therapy than needed if HCC is diagnosed later on.

Echinococcosis is a ubiquitarious, re-emergent zoonosis. Its prevalence is high in several areas of the Mediterranean region, including Southern Italy [3]. In Southern Italy, the major species of medical and public health concern are *Echinococcus granulosus *and *Echinococcus multilocularis*, which cause cystic echinococcosis and alveolar echinococcosis, respectively [3]. Hydatid disease predominantly affects liver and lungs. Both cystic echinococcosis and alveolar echinococcosis are serious diseases with a high fatality rate and poor prognosis if managed inappropriately [4,5].

Our purpose is to report a case, showing several clinical features and radiology data, which made differential diagnosis between HCC and liver echinococcosis quite challenging.

## Case presentation

A 68 year old male patient born and living in Catanzaro (Southern Italy) presented with a history of 2-month fever, dyspepsia, decreased appetite, weight loss and nicturia. Written informed consent to show the data in the case report was obtained from the patient.

He had been drinking 1 litre of wine daily and smoking 50 cigarettes, but stopped 10 years before. During the initial consultation the patient denied any chronic viral infection, including HCV. He suffered from diabetes and hypertension, so he was on insulin and anti-hypertensive therapy. On physical examination, hepatomegaly was found (stiff hepatic margin at 3 cm below the costal rim). Blood exams showed low iron levels (41 µg/dl), high alkaline phosphatase (189 IU/l), moderately raised aspartate aminotransferase (AST) level (49 IU/l) but normal alanine aminotransferase (ALT) level (31 IU/l); pseudo-cholinesterase level was 3230 IU/l, slightly lower than normal values (range 3650-12920 IU/l), bilirubin, albumin and prothrombin time where within normal values. White cell count was 9,040 cells/mm^3 ^with 1.1% eosinophils; red cells were 4,610,000/mm^3 ^and hemoglobin was 12.5 g/dl (cut off < 14 g/dl), platelets, were normal. Alpha-fetoprotein was mildly elevated at 31.50 ng/ml (cut off ≤ 8.1). No clinical and hemato-biochemical signs of cirrhosis were found.

Serology for *Salmonella spp*. (Widal), *Rickettsia spp*. (Weil Felix) and *Leishmania spp*. (IgG and IgM) were negative; serology for *Brucella spp*. showed negative Wright test and borderline IgG at 12 IU/ml (cut off ≥ 12 IU/ml). Echinococcus antibody were at borderline levels with a serum titer of 1:80 (cut off ≥1:80). Hepatitis B markers (HBcAb, HBsAg and HBsAb) were all negative. Investigation of hepatitis C virus (HCV) markers showed weakly positive HCV-antibodies, HCV-RNA = 131,000 IU/ml), and genotype 1 (gt-1).

The patient, who did not show any previous imaging exam to compare with, underwent abdominal ultrasound which revealed "a voluminous cystic mass of 11 × 7 × 9 centimeters at VII and VIII liver segments in the context of a liver with normal ultrasound structure" and the contrast-enhanced ultrasound control described "centripetal non-homogeneous impregnation of the lesion with slow and delayed wash out, due to the permanence also in the late phase (120") of either hyperechogenicity or hypoechogenicity in each contrast enhanced phase". Neither portal vein expansion nor other signs of portal hypertension were described. This finding suggested a benign lesion and a hydatid cyst (type 1 of Gharby's classification [6]) could not be excluded. Further investigations were therefore recommended.

**Figure 1:**
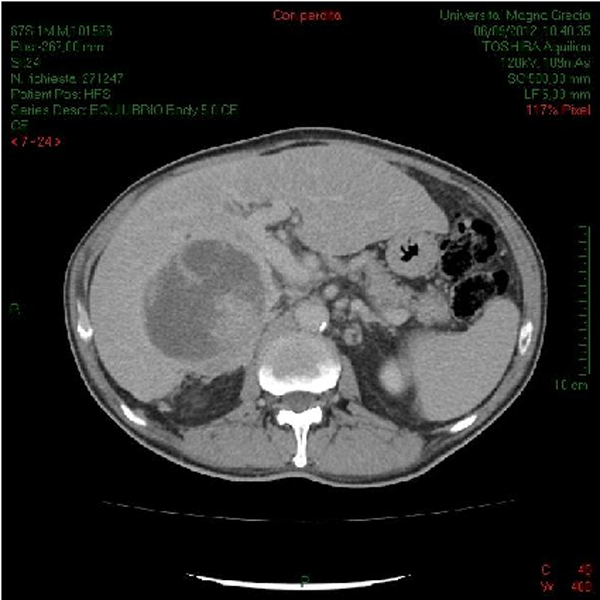
**Abdominal contrast enhanced CT scan NOTE: CT scan showed mixed hypo-isodense lesion with regular borders and enlarged lymphonodes of approximately 1.5 cm at the hepatic hilum**.

A computerized tomography (CT) scan was performed, showing that lesion was unmodified in size and describing it as "mixed hypo-isodense with regular borders", adding also the description of "enlarged lymphonodes of approximately 1.5 cm at the hepatic hilum ", and concluding for a "likely abscessual lesion" (Figure 1). However, this conclusion did not fit with blood exams, which showed normal white cell count. Moreover, this finding did not fit entirely with the typical presentation of HCC at CT, which usually appear as hypodense and, only in few cases, presents with a mixed hypo-isodense pattern [7].

Because of inconsistent data, the patient was transferred in a general surgery ward to perform liver biopsy. Due to the suspect of echinococcosis, biopsy was preceded by treatment with albendazole (to avoid peritoneal dissemination of echinococcus scolices). Notably, biopsy proved very difficult to perform, due to the presence of necrotic tissue. Eventually, the histological exam concluded for hepatocellular carcinoma.

## Conclusions

This case highlights the diagnostic difficulties to distinguish HCC from other liver lesions. Furthermore, it shows the importance of an early diagnosis of chronic HCV in order to prevent severe complications such as liver cirrhosis and HCC.

Serological markers can be useful to make a differential diagnosis between echinococcosis and HCC. However, for echinococcosis, serology can be negative in 10-15 % due to sequestration of echinococcal antigens inside the cyst, especially in case of large lesions [6,8]. In our case, the titer of *Echinococcus *antibodies (1:80) was borderline, not confirming or excluding the suspect of hydatid cyst.

Major current guidelines [9-11] suggest that alpha-fetoprotein is not diagnostic in absence of a typical radiological pattern. Moreover, in our case, alpha-fetoprotein was above the normal range but its values were only mildly elevated (31.50 ng/ml). However, this finding provided complementary information to imaging, supporting HCC diagnosis especially because the radiological pattern was inconclusive. Clearly, in such a complicated case, neither the CT scan or the haemato-biochemical results (including alpha-fetoprotein) allowed to make the definitive diagnosis that was obtained only through an invasive method (liver biopsy).

In conclusion, this case demonstrates that differential diagnosis between various lesions in the liver may be very complex and requires invasive techniques. It is therefore important to provide a timely diagnosis of late complications and treat them, as soon as possible. Clinical or biochemical features of liver cirrhosis do not necessarily precede HCC, thus they should not be invariably used to select most at risk patients. For these reasons, early diagnosis, HCV treatment, and retention into care are of outstanding importance.

## Case report #2 - Successful treatment of abbreviated Peginterferon/ribavirin in a HCV infected patient with genotype 1b. Could less be more?

Francesco Cesario

## Background

Nowadays, three drug regimens which include pegylated interferon-α2 (PEG-IFN), ribavirin (RBV) and a directly acting antiviral (DAA), namely boceprevir (BOC) and telaprevir (TPR), are the gold standard for the treatment of HCV gt-1 [12,13].

Until 2011, the gold standard was represented by dual therapy, which included PEG-IFN and RBV. At that time, the endorsed guidelines indicated that optimal duration for treatment of gt-1 and gt-4 was 48 weeks, while it was 24 weeks for treatment of gt-2 and gt-3 [14].

We present a case of a patient infected by HCV gt-1 who achieved sustained virological response (SVR) with a 24-week PEG-IFN/RBV treatment. Written informed consent to show the data in the case report was obtained from the patient.

## Case presentation

In March 2011, a 45 year old HCV gt-1b positive man was admitted to the Infectious Disease service of the "*Annunziata" *Hospital of Cosenza (Calabria region) complaining for high AST and ALT blood levels (2-3 folds higher than the upper limit of normality) and depression/anxiety. He was HIV negative, alcohol (>50 gr/day) and drug (heroin and cocaine) addicted until September 2010. Because of his depression/anxiety disorder and his drug addiction history, he was taking citalopram (20 mg/day), lorazepam (1 mg/ 3 times per day) and methadone (30 mg/day).

On physical examination, hepatomegaly was present (liver protruding 2 centimeters under the rib edge). Blood exams showed elevated AST (170 IU/l), ALT (403 IU/l) and gamma glutamyl transpeptidase (350 IU/l), low albumin (1.8 gr/dl) and platelet count (104.000 cel/µl), total and direct bilirubin were 2.0 and 1.2 mg/dl respectively. Inflammatory markers were raised (erythrocyte sedimentation rate 100 mm, C-reactive protein or 40 mg/dl). Ferritin was 540 ng/ml and hemoglobin was 11 gr/dl. Creatinine level was elevated at 1.8 mg/dl. Metavir score, estimated through liver elastography (Fibroscan) [15], was F4 (13.5 KPa). Overall, Child-Pugh score was 7 (Child A).

The patient underwent abdominal ultrasound, which revealed a slightly enlarged liver and a mass of 5 cm in the right kidney. Then, an abdominal CT scan described the renal lesion as "likely malignant".

In April 2011, the patient underwent right nephrectomy (Figure 2). A histological exam concluded for granular cell cancer, which is currently considered a malignant renal tumor [16]. Further investigations staged the tumor as T1b-N0-M0, according to the TNM staging system of the Union for International Cancer Control [17].

In May 2011, notwithstanding negative predictors of success (such as alcoholism and liver fibrosis), since HCV-RNA was low (193,873 IU/ml), patient started a dual therapy with PEG-IFN at a dosage of 1.5 μg/kg per week (120 µg per week) and RBV (1,000 mg/day). A rapid virological response (RVR) with HCV RNA undetectable at week 4 was obtained. Thus, considering RVR, concomitant medications and relevant co-morbidities, a 24-week regimen was preferred rather than the standard 48-week treatment. HCV-RNA was not detectable at week 12 (extended rapid virological response), 24 (end of treatment response) and 6 months after the end of treatment.

## Conclusions

In 2011, HCV guidelines suggested that, in patients infected by gt-1 HCV, duration of treatment could be shortened in case of RVR and low HCV viral load at baseline (<200,000 IU/ml) [14,18]. All these characteristics were present in our case and SVR was actually achieved, notwithstanding the presence of negative features, such as important co-morbidities and a difficult to treat genotype. Interestingly, after achieving RVR, rate of SVR in HCV gt-1 patients who received a 24 week long PEG-IFN/RBV treatment was 89% [18]. This case suggests that treatment duration may vary according to clinical characteristics of patients and type of virological response.

## Case series - Treatment of chronic C hepatitis in patients affected by hemoglobinopathies. Should we really improve?

Lucio Cosco, Francesco Quintieri

## Background

Patients affected by hemoglobinopathies, such as thalassemia and sickle cell disease, often develop liver fibrosis due to high iron levels [19]. Moreover, they have a high risk to acquire HCV via blood transfusions and this occurrence may lead to a worsening of the liver disease [20]. For this reason, treatment of chronic C hepatitis is important, but it may be less tolerated especially if one considers the risk of anaemia.

## Case presentations

We present a brief case series of patients (Table 1) affected by hemoglobinopathies who underwent treatment with PEG-IFN and RBV at the Infectious Diseases Service of Hospital "Pugliese-Ciaccio" in Catanzaro. Written informed consent to show the data in this case series was obtained from all patients.

**Table 1:** Characteristics of patients

Case #	Gender	Age	Hemoglobinopaty	Year of HCV diagnosis	HCV genotype	HCV-RNA (IU/l)		Hemoglobin (g/dl)		ALT (IU/l)		Metavir score	HCV therapy	Duration of therapy (weeks)	Response
3	F	35	Thalassemia mayor	1990	2a	Baseline	3,590,000	Baseline	10.1	Baseline	112	F2 (estimated through liver elastography)	Peg-Interferon 100 µg per week	24	Responder
										
						Week 4	nd	Week 4		Week 4	61				
										
						Week 12	nd	Week 12		Week 12	53				
										
						Week 24	nd	Week 24		Week 24	Normal		Ribavirin 800 mg/day		
										
						End of treatment	nd	End of treatment		End of treatment	Normal				
										
						Follow up	nd	Follow up		Follow up	Normal				

4	M	38	Thalassemia mayor	1991	4	Baseline	18,900,000	Baseline	8.9	Baseline		F2 (estimated through liver elastography)	Peg-Interferon 180 µg per week	24	Non responder
										
						Week 4	3810	Week 4		Week 4					
										
						Week 12	3830	Week 12		Week 12					
										
						Week 24	21,900	Week 24		Week 24			Ribavirin 800 mg/day		
										
						End of treatment	21,900	End of treatment		End of treatment					
										
						Follow up	3,350,000	Follow up		Follow up					

5	M	31	Drepanocytosis	1990	1b	Baseline	745,000	Baseline	8.3	Baseline	Twofold the normal	F2	Peg-Interferon 100 µg per week	72	Responder
										
						Week 4	not available	Week 4		Week 4					
										
						Week 12	34470	Week 12		Week 12					
										
						Week 24	nd	Week 24		Week 24			Ribavirin 800 mg/day		
										
						End of treatment	nd	End of treatment		End of treatment					
										
						Follow up	nd	Follow up		Follow up					

6	F	42	Thalassemia mayor	1991	2a	Baseline	3,430,000	Baseline	8.9	Baseline	106	F2 (estimated through liver elastography)	Peg-Interferon 100 µg per week	24	Relapser
										
						Week 4	nd	Week 4		Week 4					
										
						Week 12	nd	Week 12		Week 12					
										
						Week 24	nd	Week 24		Week 24			Ribavirin 800 mg/day		
										
						End of treatment	nd	End of treatment		End of treatment					
										
						Follow up	1,360,000	Follow up		Follow up					

a) Case ***#***3 - In October 2010, a 35 year old woman was admitted to the "*Pugliese-Ciaccio" *Hospital to start HCV treatment. She was affected by thalassemia major and she knew to be HCV positive since 1990. She had HCV-RNA of 3,590,000 IU/l and gt-2a. ALT was 112 IU/l and hemoglobin was 10.1 g/dl. Liver Metavir score, estimated via transient elastography (Fibroscan™) [15], was F2. She started a 24-week treatment with PEG-IFNα2b (100 µg per week) and RBV (800 mg/day). RBV dosage was maintained until the end of treatment. Next controls showed not detectable HCV-RNA at weeks 4, 12 and 24. ALT decreased to 61 IU/ml by week 4, while it was normal at week 24. After 2 years, HCV-RNA was still not detectable (SVR) and ALT was normal. The patient was also treated with blood transfusions, which increased from a transfusion/month before HCV treatment to a transfusion/10 days during PEG-IFN/RBV therapy. No hemolytic crisis occurred.

b) Case ***#***4 - In November 2011, a 38 year old, HCV positive and thalassemic (thalassemia major) man started therapy with PEG-IFNα2a (180 µg per week) and RBV (800 mg/day). His history of HCV had started in 1991. He was infected by HCV gt-4 and, at baseline, HCV-RNA was 18,900,000 IU/l. Moderate fibrosis resulted from liver elastography (7.9 KPa) with an estimated Metavir Score of F2 [15]. Blood exams showed anaemia (hemoglobin 8.9 g/dl). The scheduled duration of treatment was 48 weeks but, unfortunately, treatment was stopped at week 24 because of non response. Indeed, HCV-RNA was detectable at week 4 (3810 IU/l), 12 (3830 IU/l) and 24 (21,900 IU/l). Dosage of RBV was kept at 800 mg/day and no severe hemolysis occurred. After 7 months from the suspension of therapy, viral load was 3,350,000 IU/l. During treatment, the patient received an higher number of blood transfusions (a transfusion/2 weeks) than before treatment (around a transfusion/3 weeks).

c) Case ***#***5 - In December 2011, a 31 year old man came to the Infectious Diseases Service of Hospital "Pugliese-Ciaccio" to start HCV treatment. He was affected by drepanocytosis (sickle cell disease) and infected by HCV gt -1b since 1990. At baseline, HCV-RNA was 745,000 IU/l. Mild chronic hepatitis was revealed by liver biopsy (Metavir score F2). ALT was twofold the normal value and hemoglobin was 8.3 g/dl. He underwent a 18 month long therapy, from December 2011 to June 2013, with PEG-IFNα2b (100 µg per week) and RBV (800 mg/day). HCV-RNA was 34,470 IU/l at week 12 and it became not detectable at week 24. At end of treatment HCV-RNA was not detectable and ALT was normal. Due to the absence of severe hemolytic crisis, increasing the number of blood transfusions (from 1/3 weeks to 1/10 days ) was the only intervention required to manage anaemia, without any needs to reduce RBV.

d) Case ***#***6 - In March 2012, a 42 year old woman started PEG-IFN/RBV therapy. She was affected by thalassemia major and she knew to be HCV gt-2a positive since 1991. Blood exams showed anaemia (hemoglobin 8.9 g/dl) and elevated ALT (106 IU/l). The HCV viral load was 3,430,000 IU/l. Liver stiffness (assessed through Fibroscan™) was 8.5 KPa (Metavir score F2) [15]. She followed treatment for 24 weeks with PEG-IFNα2b (100 µg per week) and RBV (800 mg/day). She reached RVR and end of treatment response, included the normalization of ALT. Unfortunately, 5 months after the end of therapy, she relapsed (HCV-RNA 1,360,000 IU/l). Blood transfusions were performed during treatment more frequently than before therapy (from a transfusion/3 weeks to a transfusion/week). However, reduction of RBV was not necessary and hemolytic crisis did not appear during PEG-IFN/RBV.

## Conclusions

Patients with hemoglobinopathies often exhibit significant liver fibrosis [19]. However, in all of our cases, liver disease, estimated through liver elastography, was moderate (corresponding to F2 at Metavir score). It has to be seen whether elastography is an accurate method to estimate fibrosis in presence of liver damage associated with hemoglobinopathies beside HCV. Interestingly, before HCV treatment, all patients presented with high ALT levels that normalized by the end of treatment. This suggests that, in case of moderate fibrosis, HCV treatment reduces liver inflammation even when SVR is not achieved.

Anaemia is the most common side effect of PEG-IFN/RBV and patients with hemoglobinopathies require a higher number of blood transfusions when they are treated with PEG-IFN/RBV [21]. Indeed, in our short case series, all patients needed more blood transfusions to overcome reduction of hemoglobin. Moreover, anaemia is the most common side effect of DAAs, which represent the current gold standard for treatment of gt-1 HCV in association with PEG-IFN/RBV. Due to the elevated risk of severe anaemia, current available triple therapy with PEG-IFN/RBV/DAA may be difficult to apply in these patients. Consequently, we are looking forward to the next generation DAAs, which are expected to be strongly effective even in absence of PEG-IFN and RBV.

## List of abbreviations

HCV, hepatitis C virus; gt, genotype; HCC, hepatocellular carcinoma; AST, aspartate aminotransferase; ALT alanine aminotransferase; CEUS, contrast- enhanced ultrasound;CT, computerized tomography; HBV, hepatitis B virus; PEG-IFN, pegylated interferon; RBV, ribavirin; DAA, directly acting antiviral; BOC, boceprevir; TPR telaprevir; SVR, sustained viralogical response; RVR, rapid viral response.

## Competing interests

The authors declare that they have no competing interests.

## Authors' contributions

AS contributed to manuscript writing; GM contributed to manuscript writing and manuscript revision; SVM, VV contributed to manuscript writing; VP, CC, FM, PD, LC, FQ, FC collected clinical data; MCL contributed to manuscript revision; AG collected laboratory data; CT contributed to manuscript writing and manuscript revision; AF contributed to manuscript revision.

## Declarations

The publication of this supplement was partly supported by an unrestricted grant provided by Roche. The articles were independently prepared by the authors with no input from Roche. Roche were not involved in selecting the articles for the supplement. The pegylated IFN/pegIFN treatment mentioned in this article is produced by Roche.

This article has been published as part of *BMC Infectious Diseases *Volume 14 Supplement 5, 2014: Proceedings of the Third Workshop of the HCV Study Group in the Calabria Region: Results from the South Italian Network for Rational Guidelines and International Epidemiology (SINERGIE) Project. The full contents of the supplement are available online at http://www.biomedcentral.com/bmcinfectdis/supplements/14/S5.
